# Supplementary material for: Intraspecific variation in the cochleae of harbour porpoises (Phocoena phocoena) and its implications for comparative studies across odontocetes
Source: PeerJ. 2020 Apr 13;8:e8916. doi: 10.7717/peerj.8916 (PMC7161573; doi:10.7717/peerj.8916)
Supplement: Supplemental Information 2 — Table S1. Specimen accession numbers and museum of origin. AMNH = American Museum of Natural History, New York, USA; IRSNB = Royal Belgian Institute of Natural Sciences; QMJM = Queensland Museum; NHMUK = Natural History Museum, London; NMVC = Museum Victoria, Melbourne, Australia; NMB = Naturhistorisches Museum, Basel, Switzerland. Side refers to the side of the head the cochlea came from. Table S2. Scan Parameters of newly scanned specimens of Phocoena phocoena at the NHM. NHMUK = Natural History Museum, London. All specimens used a scan power (kV) of 100 and 1,999 slices. Details for all other specimens can be found in (Park et al., 2019). Table S3. Additional information on Phocoena phocoena specimens. Side refers to the side of the head the cochlea came from. CBL is condylobasal length (skull length) recorded in mm. Note that not all cochleae had a corresponding skull in the collections Table S6. Summary of coefficients of variation (CV) found in other studies of odontocete cochlea. Where these studies contained right and left cochleae from one individual we first took the mean for the individual, then calculated CV across individuals.W2, Width of basal turn perpendicular to cochlear width; ITD, Inter-turn distance, maximum distance between turns; FC, area of fenestra cochlearis. * This value is based on measurements from right and left cochlea of 12 individuals (i.e., 24 cochlea) rather than the mean value for each individual because only overall means are published in the paper. Table S7: Results of ANOVAs using principal components (PCs) accounting for 95% of the variance as response variables, and whether a specimen was Phocoena phocoena or another odontocete species as the explanatory variable. 3D shape data analyses used PCs 1 to 26; linear measurements analyses used PCs 1 to 6. Significant p values (p < 0.05) are in bold. Table S8. Results of MANOVA analyses using principal components (PCs 1 to 26) that account for 95% of the variance in 3D shape as the r [file peerj-08-8916-s002.docx]

**Supplemental Information from: *Intraspecific variation in the cochleae of harbour porpoises (Phocoena phocoena) and its implications for comparative studies across odontocetes.***

Maria Clara Iruzun Martins, Travis Park, Rachel Racicot and Natalie Cooper

*Table S1. Specimen accession numbers and museum of origin. AMNH = American Museum of Natural History, New York, USA; IRSNB* = Royal Belgian Institute of Natural Sciences; *QMJM* = Queensland Museum*; NHMUK = Natural History Museum, London; NMVC = Museum Victoria, Melbourne, Australia; NMB= Naturhistorisches Museum, Basel, Switzerland. Side refers to the side of the head the cochlea came from.*

| **Specimen number** | **Side** | **Taxon** |
| --- | --- | --- |
| NHMUK_CE1982.315 | Right | *Berardius arnuxii* |
| NHMUK_CE1952.6.20.4 | Right | *Cephalorhynchus commersonii* |
| NHMUK_CE1948.7.27.1 | Right | *Cephalorhynchus heavisidii* |
| NMBCIII1086 | Right | *Delphinapterus leucas* |
| NMB6679 | Right | *Delphinus delphis* |
| NHMUK_CE1874.11.25.1 | Right | *Feresa attenuata* |
| NHMUK_CE1947.12.31.4 | Right | *Globicephala melas* |
| NHMUK_CE1920.12.16.1 | Right | *Grampus griseus* |
| NHMUK_CE1862.12.2.2 | Right | *Hyperoodon ampullatus* |
| NHMUK1934.7.23.3 | Left | *Hyperoodon planifrons* |
| NMB7167 | Right | *Inia geoffrensis* |
| NMVC24976 | Left | *Kogia breviceps* |
| NHMUK_CE1952.8.28.1 | Right | *Kogia sima* |
| NHMUK_CE1895.5.9.1 | Right | *Lagenodelphis hosei* |
| NHMUK_CESW1928.19 | Right | *Lagenorhynchus acutus* |
| NHMUK_CE1848.7.12.12 | Right | *Lagenorhynchus albirostris* |
| NHMUK_CE1944.11.30.1 | Right | *Lagenorhynchus australis* |
| NHMUK_CE1960.8.24.1 | Right | *Lagenorhynchus cruciger* |
| NHMUK_CE1966.10.25.1 | Right | *Lagenorhynchus obliquidens* |
| NHMUK_CE1841.1733 | Left | *Lagenorhynchus obscurus* |
| AMNH57333 | Left | *Lipotes vexillifer* |
| IRSNB16232 | Right | *Mesoplodon bidens* |
| NMVC31378 | Left | *Mesoplodon grayi* |
| NHMUK_CE1876.2.16.3 | Right | *Mesoplodon hectori* |
| NHMUK_CE1920.5.20.1 | Right | *Mesoplodon mirus* |
| AMNH73315 | Left | *Monodon monoceros* |
| NHMUK_CE1903.9.12.3 | Right | *Neophocaena phocaenoides* |
| NHMUK_CE1883.11.20.2 | Right | *Orcaella brevirostris* |
| QMJM4700 | Right | *Orcaella heinsohni* |
| NHMUK_CESW1927.28 | Right | *Orcinus orca* |
| NHMUK_CE1992.100 | Right | *Peponocephala electra* |
| NHMUK_CE1939.9.30.1 | Right | *Phocoena dioptrica* |
| NHM_SW1954.15 | Left | *Phocoena phocoena* |
| NHMUK_1846.12.15.6 | Left | *Phocoena phocoena* |
| NHMUK_1873.6.3.45 | Left | *Phocoena phocoena* |
| NHMUK_92.265 | Left | *Phocoena phocoena* |
| NHMUK_1925.4.29.1 | Left | *Phocoena phocoena* |
| NHMUK_1933.15 | Left | *Phocoena phocoena* |
| NHMUK_1933.17 | Right | *Phocoena phocoena* |
| NHMUK_1934.45 | Left | *Phocoena phocoena* |
| NHMUK_1934.51 | Left | *Phocoena phocoena* |
| NHMUK_1965.3.30.1 | Left | *Phocoena phocoena* |
| NHMUK_1992.263 | Left | *Phocoena phocoena* |
| NHMUK_SW1926.29 | Right | *Phocoena phocoena* |
| NHMUK_SW1934.14 | Left | *Phocoena phocoena* |
| NHMUK_SW1934.31 | Left | *Phocoena phocoena* |
| NHMUK_SW1934.32 | Left | *Phocoena phocoena* |
| NHMUK_SW1937.24 | Left | *Phocoena phocoena* |
| NMB10131 | Right | *Phocoena phocoena* |
| NMVC27654 | Left | *Phocoena phocoena* |
| *Phocoena_sinus* | Right | *Phocoena sinus* |
| IRSNB_21219 | Right | *Phocoena spinipinnis* |
| NHMUK_CE1965.1.19.2 | Right | *Phocoenoides dalli* |
| NHMUK_CE893 | Left | *Physeter macrocephalus* |
| NMVC27417.2 | Right | *Platanista gangetica* |
| MNHN1934.375 | Right | *Pontoporia blainvillei* |
| NHMUK_CE1992.248 | Right | *Pseudorca crassidens* |
| NHMUK_CE1856.8.2.2 | Right | *Sotalia fluviatilis* |
| IRSNB_20137 | Left | *Sotalia guianensis* |
| NHMUK_CE1914.1.14.1 | Right | *Sousa chinensis* |
| NHMUK_CE1893.8.1.1 | Right | *Sousa teuszii* |
| NHMUK_CE1990.98 | Right | *Stenella attenuata* |
| NHMUK_CE1940.3.2.1 | Right | *Stenella coeruleoalba* |
| NHMUK_CE1990.104 | Right | *Stenella longirostris* |
| NMVC36961 | Left | *Steno bredanensis* |
| NMVC37967.6 | Right | *Tasmacetus shepherdi* |
| NHMUK CE1882.1.2.3 | Right | *Tursiops aduncus* |
| NHMUK_CE1866.8.7.1 | Right | *Tursiops truncatus* |
| NHMUK_CE1915.7.20.1 | Right | *Ziphius cavirostris* |

*Table S2. Scan Parameters of newly scanned specimens of Phocoena phocoena at the NHM. NHMUK = Natural History Museum, London. All specimens used a scan power (kV) of 100 and 1999 slices. Details for all other specimens can be found in Park et al. 2019.*

| **Specimen number** | **Voxel Size (μm)** |
| --- | --- |
| NHMUK_1846.12.15.6 | 53.40 |
| NHMUK_1873.6.3.45 | 49.38 |
| NHMUK_1925.4.29.1 | 48.73 |
| NHMUK_SW1926.29 | 49.69 |
| NHMUK_1992.265 | 49.69 |
| NHMUK_SW1954.15 | 49.38 |
| NHMUK_1965.3.30.1 | 48.73 |
| NHMUK_1992.263 | 48.31 |
| NHMUK_SW1934.32 | 48.73 |
| NHMUK_SW1933.17 | 45.97 |
| NHMUK_1934.31 | 53.40 |
| NHMUK_SW1934.14 | 48.83 |
| NHMUK_SW1934.45 | 48.31 |
| NHMUK_SW1933.15 | 45.97 |
| NHMUK_SW1934.51 | 48.83 |
| NHMUK_SW1937.24 | 48.73 |

*Table S3. Additional information on Phocoena phocoena specimens. Side refers to the side of the head the cochlea came from. CBL is condylobasal length (skull length) recorded in mm. Note that not all cochleae had a corresponding skull in the collections.*

| **Specimen number** | **Side** | **Sex** | **Origin** | **CBL** |
| --- | --- | --- | --- | --- |
| NHMUK_1846.12.15.6 | Left | - | UK | 241 |
| NHMUK_1873.6.3.45 | Left | - | USA | - |
| NHMUK_1892.265 | Left | - | UK | - |
| NHMUK_1925.4.29.1 | Left | Male | UK | - |
| NHMUK_1933.15 | Left | - | UK | 245 |
| NHMUK_1933.17 | Right | Male | UK | - |
| NHMUK_1934.45 | Left | Male | UK | - |
| NHMUK_1934.51 | Left | - | UK | 251 |
| NHMUK_1965.3.30.1 | Left | Female | UK | 259 |
| NHMUK_1992.263 | Left | - | UK | - |
| NHMUK_SW1926.29 | Right | Female | UK | 271 |
| NHMUK_SW1934.14 | Left | Female | UK | 254 |
| NHMUK_SW1934.31 | Left | Male | UK | - |
| NHMUK_SW1934.32 | Left | Male | UK | 235 |
| NHMUK_SW1937.24 | Left | Female | UK | 251 |
| NHMUK_SW1954.15 | Left | - | UK | - |
| NMB10131 | Right | - | UK | - |
| NMVC27654 | Left | - | UK | 251 |

**Tables S4 and S5 are too large to include here so can be found as a separate Excel file. These tables include ecological data on each species and a reference list for where these data came from.**

*Table S6. Summary of coefficients of variation (CV) found in other studies of odontocete cochlea. Where these studies contained right and left cochleae from one individual we first took the mean for the individual, then calculated CV across individuals.* *W2 = Width of basal turn perpendicular to cochlear width; ITD = Inter-turn distance, maximum distance between turns; FC = area of fenestra cochlearis.* **This value is based on measurements from right and left cochlea of 12 individuals (i.e. 24 cochlea) rather than the mean value for each individual because only overall means are published in the paper.*

| **Reference** | **Species** | **Number of individuals** | **Number of cochleae** | **Measurement** | **CV (%)** |
| --- | --- | --- | --- | --- | --- |
| Costeur et al, 2018 | *Pontoporia blainvillei* | 4 | 4 | Number of turns | 0.00 |
| Costeur et al, 2018 | *Pontoporia blainvillei* | 4 | 4 | Cochlear height | 3.35 |
| Costeur et al, 2018 | *Pontoporia blainvillei* | 4 | 4 | Axial pitch | 3.52 |
| Costeur et al, 2018 | *Pontoportia sp* | 4 | 4 | Number of turns | 0.00 |
| Costeur et al, 2018 | *Pontoportia sp* | 4 | 4 | Cochlear height | 2.04 |
| Costeur et al, 2018 | *Pontoportia sp* | 4 | 4 | Axial pitch | 2.04 |
| Costeur et al, 2018 | *Ziphiidae sp* | 2 | 2 | Number of turns | 9.43 |
| Costeur et al, 2018 | *Ziphiidae sp* | 2 | 2 | Cochlear height | 13.99 |
| Costeur et al, 2018 | *Ziphiidae sp* | 2 | 2 | Axial pitch | 23.33 |
| Costeur et al, 2018 | *Inia geoffrensis* | 2 | 2 | Number of turns | 0.00 |
| Costeur et al, 2018 | *Inia geoffrensis* | 2 | 2 | Cochlear height | 10.88 |
| Costeur et al, 2018 | *Inia geoffrensis* | 2 | 2 | Axial pitch | 10.95 |
| Schnitzler et al, 2017 | *Physeter macrocephalus* | 12 | 24 | Cochlear height | 4.01* |
| Schnitzler et al, 2017 | *Physeter macrocephalus* | 12 | 24 | Cochlear width | 3.50* |
| Schnitzler et al, 2017 | *Physeter macrocephalus* | 12 | 24 | Cochlear canal length | 4.39* |
| Racicot et al, 2016 | *Phocoena sinus* | 2 | 2 | Cochlear volume | 10.23 |
| Racicot et al, 2016 | *Phocoena sinus* | 2 | 2 | Cochlear canal length | 1.55 |
| Racicot et al, 2016 | *Phocoena sinus* | 2 | 2 | Number of turns | 0.39 |
| Racicot et al, 2016 | *Phocoena sinus* | 2 | 2 | Basal ratio | 0.00 |
| Racicot et al, 2016 | *Phocoena sinus* | 2 | 2 | Cochlear height | 3.48 |
| Racicot et al, 2016 | *Phocoena sinus* | 2 | 2 | Cochlear width | 3.91 |
| Racicot et al, 2016 | *Phocoena sinus* | 2 | 2 | Axial pitch | 3.84 |
| Racicot et al, 2018 | *Delphinapterus leucas* | 2 | 3 | Cochlear canal length | 2.39 |
| Racicot et al, 2018 | *Delphinapterus leucas* | 2 | 3 | Cochlear width | 2.88 |
| Racicot et al, 2018 | *Delphinapterus leucas* | 2 | 3 | Cochlear height | 10.52 |
| Racicot et al, 2018 | *Delphinapterus leucas* | 2 | 3 | W2 | 3.67 |
| Racicot et al, 2018 | *Delphinapterus leucas* | 2 | 3 | ITD | 13.95 |
| Racicot et al, 2018 | *Delphinapterus leucas* | 2 | 3 | FC | 3.65 |
| Racicot et al, 2018 | *Delphinapterus leucas* | 2 | 3 | Number of turns | 5.59 |
| Racicot et al, 2018 | *Monodon monocerus* | 5 | 9 | Cochlear canal length | 5.28 |
| Racicot et al, 2018 | *Monodon monocerus* | 5 | 9 | Cochlear width | 3.16 |
| Racicot et al, 2018 | *Monodon monocerus* | 5 | 9 | Cochlear height | 6.08 |
| Racicot et al, 2018 | *Monodon monocerus* | 5 | 9 | W2 | 1.42 |
| Racicot et al, 2018 | *Monodon monocerus* | 5 | 9 | ITD | 11.30 |
| Racicot et al, 2018 | *Monodon monocerus* | 5 | 9 | FC | 16.03 |
| Racicot et al, 2018 | *Monodon monocerus* | 5 | 9 | Number of turns | 1.95 |

*Table S7: Results of ANOVAs using principal components (PCs) accounting for 95% of the variance as response variables, and whether a specimen was Phocoena phocoena or another odontocete species as the explanatory variable. 3D shape data analyses used PCs 1 to 26; linear measurements analyses used PCs 1 to 6.* Significant p values (p < 0.05) are in bold.

| **3D shape data** | | | | |
| --- | --- | --- | --- | --- |
| **PC** | **df** | **F** | **p value** | **Bonferroni corrected p value** |
| PC1 | 1,67 | 10.44 | **0.002** | **0.050** |
| PC2 | 1,67 | 0.316 | 0.576 | - |
| PC3 | 1,67 | 27.44 | **< 0.001** | **< 0.001** |
| PC4 | 1,67 | 2.151 | 0.147 | - |
| PC5 | 1,67 | 2.582 | 0.113 | - |
| PC6 | 1,67 | 2.225 | 0.140 | - |
| PC7 | 1,67 | 0.617 | 0.435 | - |
| PC8 | 1,67 | 0.057 | 0.812 | - |
| PC9 | 1,67 | 1.546 | 0.218 | - |
| PC10 | 1,67 | 4.209 | 0.044 | - |
| PC11 | 1,67 | 2.819 | 0.098 | - |
| PC12 | 1,67 | 1.723 | 0.194 | - |
| PC13 | 1,67 | 0.319 | 0.574 | - |
| PC14 | 1,67 | 1.327 | 0.253 | - |
| PC15 | 1,67 | 0.094 | 0.760 | - |
| PC16 | 1,67 | 2.989 | 0.088 | - |
| PC17 | 1,67 | 0.046 | 0.830 | - |
| PC18 | 1,67 | 0.408 | 0.525 | - |
| PC19 | 1,67 | 0.202 | 0.655 | - |
| PC20 | 1,67 | 0.850 | 0.360 | - |
| PC21 | 1,67 | 3.345 | 0.072 | - |
| PC22 | 1,67 | 0.235 | 0.629 | - |
| PC23 | 1,67 | 0.446 | 0.506 | - |
| PC24 | 1,67 | 0.077 | 0.782 | - |
| PC25 | 1,67 | 0.074 | 0.786 | - |
| PC26 | 1,67 | 0.286 | 0.594 | - |
| **Linear measurements** | | | | |
| **PC** | **df** | **F** | **p value** | **Bonferroni corrected p value** |
| PC1 | 1,67 | 24.303 | **< 0.001** | **< 0.001** |
| PC2 | 1,67 | 8.237 | **0.005** | **0.033** |
| PC3 | 1,67 | 0.088 | 0.767 | - |
| PC4 | 1,67 | 5.139 | **0.027** | 0.16 |
| PC5 | 1,67 | 1.117 | 0.294 | - |
| PC6 | 1,67 | 0.229 | 0.634 | - |

*Table S8. Results of MANOVA analyses using principal components (PCs 1 to 26) that account for 95% of the variance in 3D shape as the response variable, and whether a specimen was Phocoena phocoena or another odontocete species (group), and additional taxonomic or ecological variables (various) as the explanatory variables.*

| **analysis** | **variable** | **df** | **F/approx F** | **Pillai** | **p** |
| --- | --- | --- | --- | --- | --- |
| Family | group | 1,58 | 5.964 | 0.921 | < 0.001 |
|  | family | 9,58 | 1.671 | 2.923 | < 0.001 |
| Habitat | group | 1,59 | 6.171 | 0.920 | < 0.001 |
|  | habitat | 4,59 | 1.671 | 2.923 | 0.003 |
| Diet | group | 1,60 | 6.433 | 0.919 | < 0.001 |
|  | diet | 3,60 | 1.714 | 2.207 | 0.007 |
| Feeding mode | group | 1,60 | 6.357 | 0.918 | < 0.001 |
|  | feeding | 3,60 | 0.927 | 1.803 | 0.647 |
| Dive type | group | 1,60 | 6.768 | 0.923 | < 0.001 |
|  | dive type | 3,60 | 1.822 | 2.242 | 0.003 |
| Hearing type | group | 1,66 | 6.309 | 0.898 | < 0.001 |
|  | hearing type | 1,66 | 3.572 | 0.833 | < 0.001 |

*Table S9. Results of Procrustes MANOVA and standard MANOVA analyses using either Procrustes aligned coordinates* *or principal components (PCs) accounting for 95% of the variance as the response variable, and whether a specimen was Phocoena phocoena or another phocoenid species as the explanatory variable. 3D shape data analyses used PCs 1 to 14; linear measurements analyses used PCs 1 to 7. Significant p values (< 0.05) are in bold.*

| **3D shape data** | | | | |
| --- | --- | --- | --- | --- |
| **test** | **df** | **F/approx F** | **Pillai** | **p** |
| Procrustes ANOVA | 1,19 | 2.958 | NA | **0.026** |
| MANOVA | 1,19 | 3.398 | 0.888 | 0.070 |
| **Linear measurements** | | | | |
| MANOVA | 1,19 | 3.155 | 0.629 | **0.035** |

*Table S10. Possible correlates of intraspecific variation in the cochleae of Phocoena phocoena specimens. Results are from MANOVAs using principal components (PCs) accounting for 95% of the variance in the cochleae shape as the response variable, and either the side of the head the cochlea came from, the sex, origin (UK or USA), or log skull length (condylobasal length in mm) of the specimens, as the predictor variables. 3D shape data analyses used PCs 1 to 16; linear measurements analyses used PCs 1 to 7.*

| **3D shape data** | | | | | |
| --- | --- | --- | --- | --- | --- |
| **predictor** | **df** | **approx F** | **Pillai** | **p** | **bonferroni** |
| side | 1,16 | 1.837 | 0.896 | 0.340 | 1.000 |
| sex*^†^* | 1,7 | 1.114 | 0.770 | 0.544 | 1.000 |
| origin | 1,16 | 10.69 | 0.980 | **0.038** | 0.152 |
| skull length*^†^* | 1,7 | 19.41 | 0.983 | 0.050 | 1.000 |
| **Linear measurements** | | | | | |
| side | 1,16 | 0.571 | 0.285 | 0.765 | 1.000 |
| sex | 1,7 | 6.613 | 0.979 | 0.291 | 1.000 |
| origin | 1,16 | 0.376 | 0.861 | 0.566 | 1.000 |
| skull length | 1,7 | 4.226 | 0.967 | 0.359 | 1.000 |

*^†^For sex and condylobasal length we have fewer specimens with data (n = 9 for each) so there are not enough observations to fit MANOVAs of all 16 PCs. In these analyses we therefore use only PC1 to PC6, which accounted for 75% of the variance in the cochlear shape.*

*Table S11. Results of Procrustes MANOVA and standard MANOVA analyses using either Procrustes aligned coordinates* *or principal components (PCs) accounting for 95% of the variance as the response variable, and whether a specimen was Phocoena phocoena or another odontocete species as the explanatory variable, but excluding the USA P.phocoena specimen (NHMUK_1873.6.3.45).* *3D shape data analyses used PCs 1 to 26; linear measurements analyses used PCs 1 to 6. Significant p values (< 0.05) are in bold.*

| **3D shape data** | | | | |
| --- | --- | --- | --- | --- |
| **test** | **df** | **F/approx F** | **Pillai** | **p** |
| Procrustes ANOVA | 1,66 | 5.374 | NA | **0.001** |
| MANOVA | 1,66 | 8.376 | 0.842 | **< 0.001** |
| **Linear measurements** | | | | |
| MANOVA | 1,66 | 8.570 | 0.457 | **< 0.001** |

**Supplemental Figures**


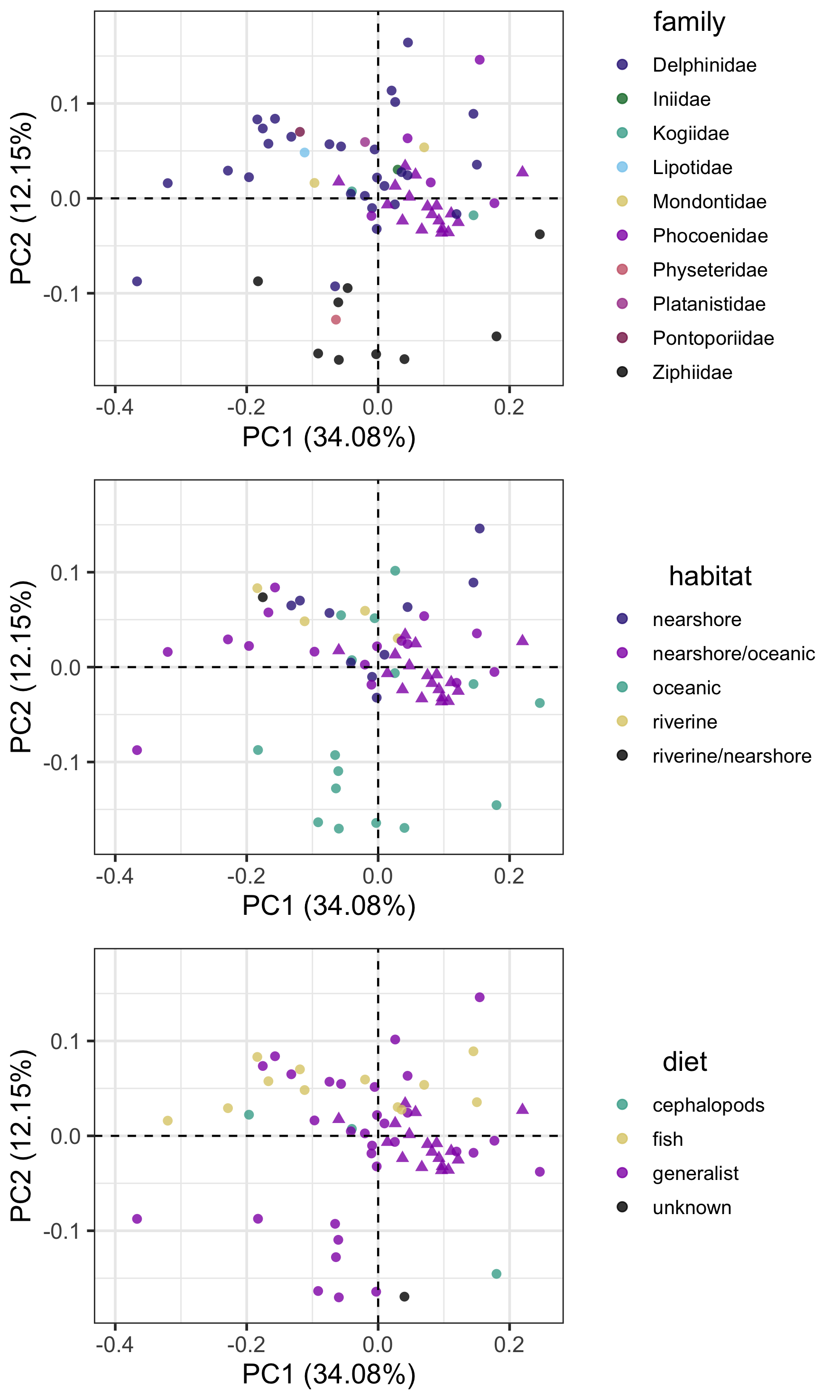


*Figure S1: Principal components plots of PC1 and PC2 for 3D cochlea shape data, coloured according to taxonomic family, habitat or diet. Triangles are Phocoena phocoena specimens; circles are all other odontocete species. numbers in brackets show the percentage variance explained for each PC.*

*
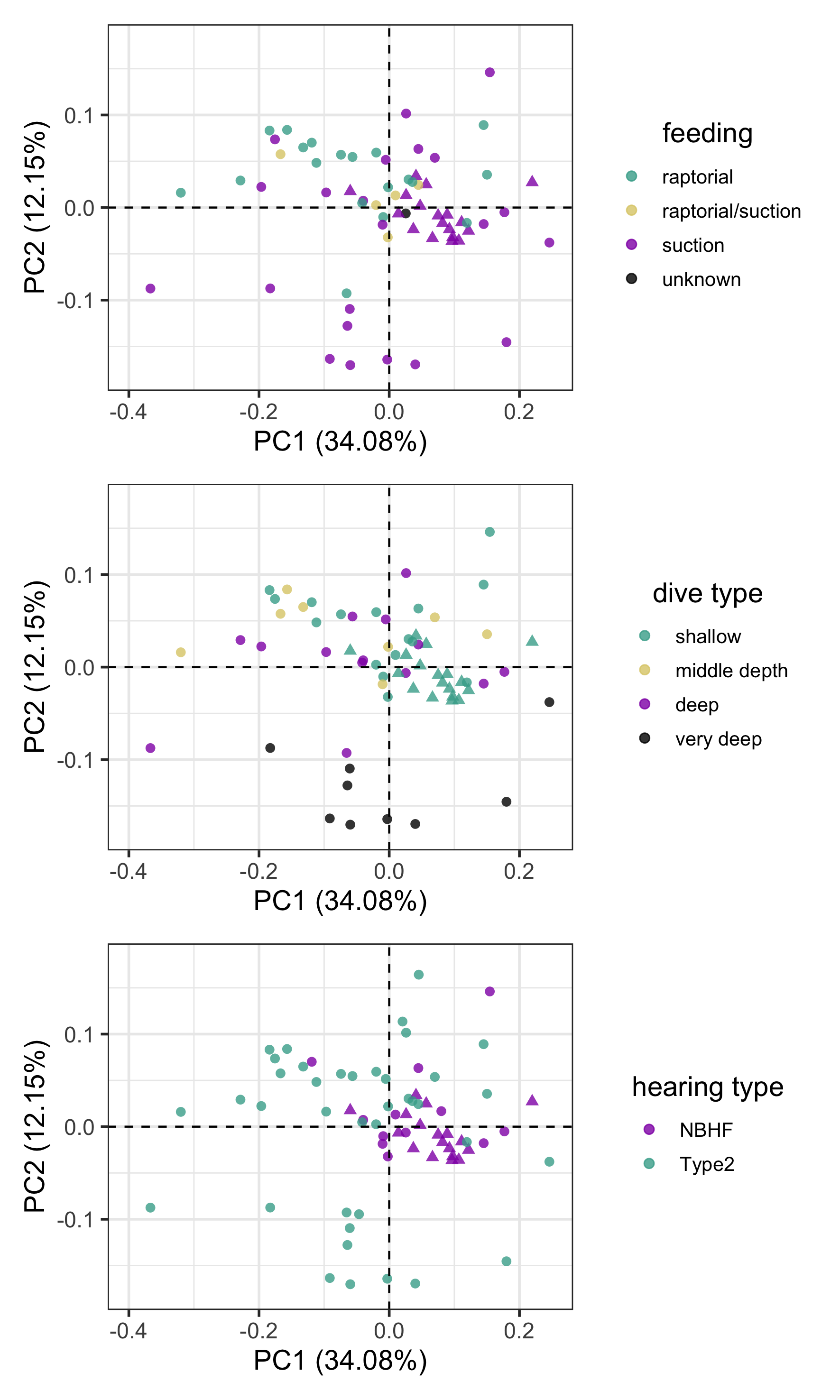
*

*Figure S2: Principal components plots of PC1 and PC2 for 3D cochlea shape data, coloured according to taxonomic feeding mode, dive type or hearing type. Triangles are Phocoena phocoena specimens; circles are all other odontocete species. numbers in brackets show the percentage variance explained for each PC.*


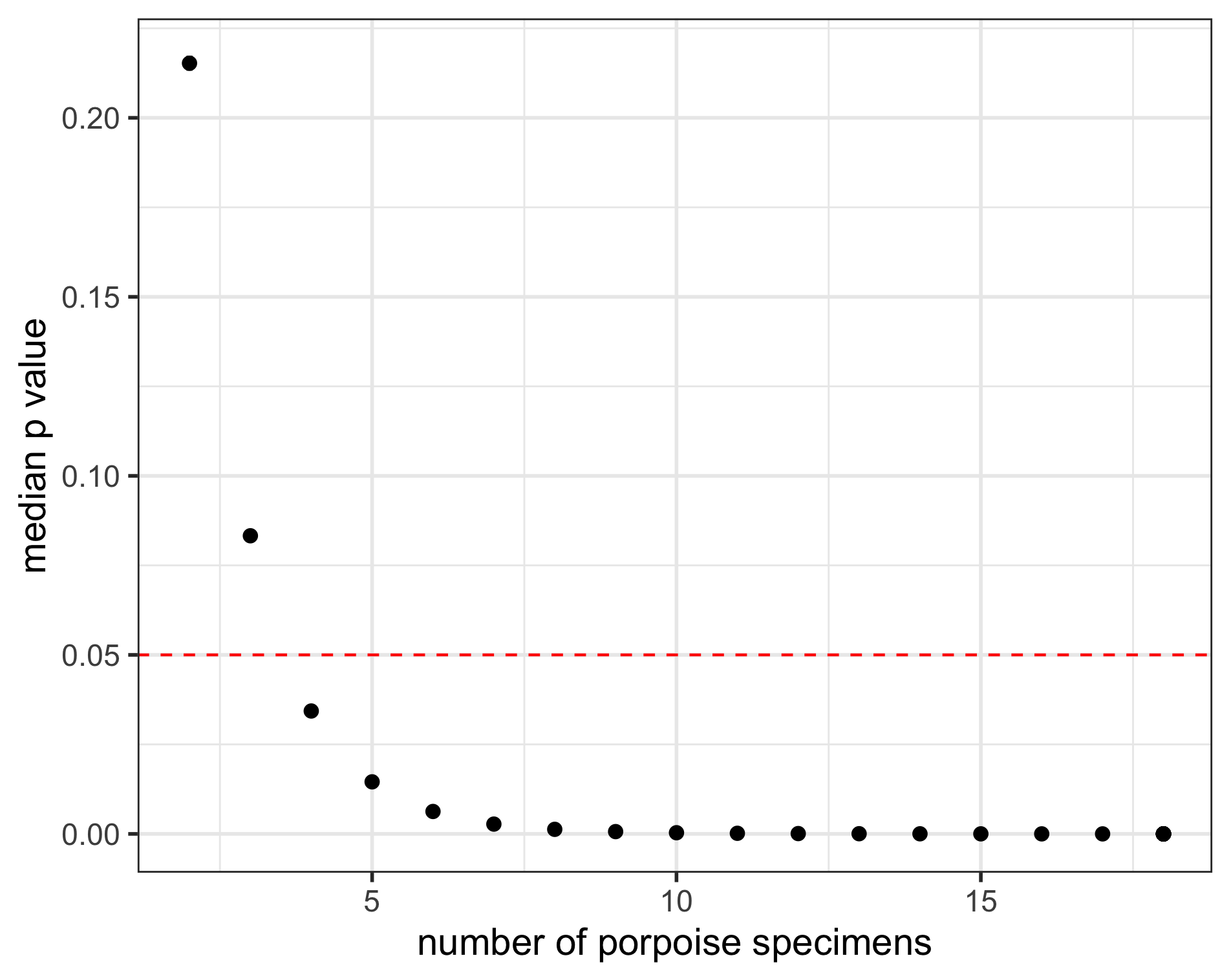


*Figure S3: Median p values from multivariate analyses of variance (MANOVA) on principal components (PCs) of 3D cochlea shape data with varying numbers of Phocoena phocoena specimens included. For each number of Phocoena phocoena specimens we ran the analyses for each possible combination of specimens. Significance at p < 0.05 is indicated by the red dashed line.*

*
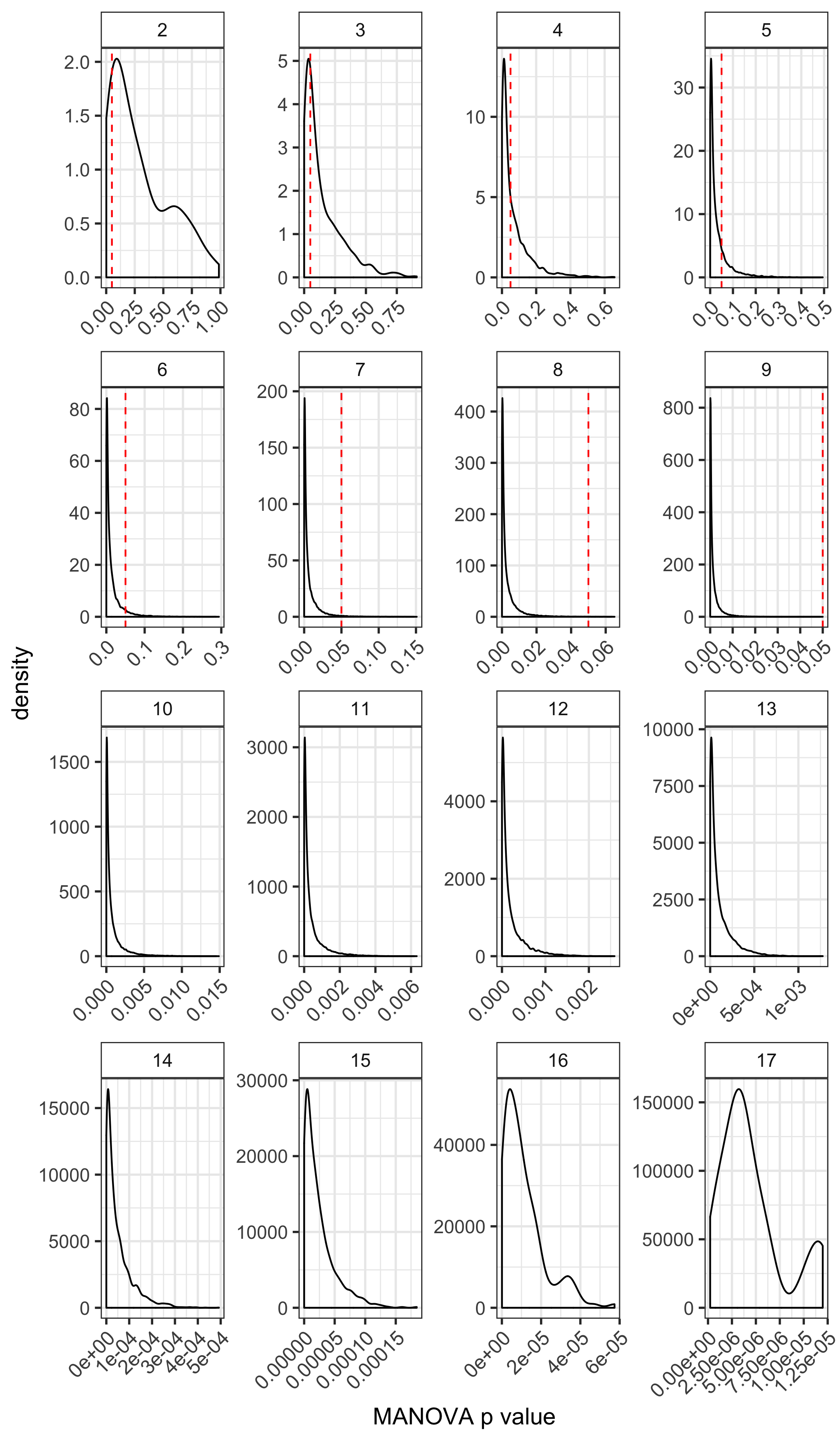
*

*Figure S4: Kernal density plots showing p values from multivariate analyses of variance (MANOVA) on principal components (PCs) of 3D cochlea shape data with varying numbers of Phocoena phocoena specimens included. For each number of Phocoena phocoena specimens we ran the analyses for each possible combination of specimens. Significance at p < 0.05 is indicated by the red dashed line, which is not visible for n > 9.*

**References**

Park, T., Mennecart, B., Costeur, L., Grohé., and Cooper, N. 2019. Convergent evolution in toothed whale cochleae. *BMC Evolutionary Biology*. 9, 195.
